# *Pickpocket315* affects male mating behavior in the yellow fever mosquito *Aedes aegypti*

**DOI:** 10.1093/g3journal/jkaf297

**Published:** 2025-12-10

**Authors:** Claudia A S Wyer, I Alexandra Amaro, Sylvie Pitcher, Alongkot Ponlawat, Laura C Harrington, Mariana F Wolfner, Brian Hollis, Lauren J Cator

**Affiliations:** Department of Life Sciences, Imperial College London, Ascot SL5 7PY, United Kingdom; Department of Entomology, Cornell University, Ithaca, NY 14853, United States; Department of Entomology, Cornell University, Ithaca, NY 14853, United States; Department of Entomology, Cornell University, Ithaca, NY 14853, United States; Department of Entomology, Armed Forces Research Institute of Medical Sciences, Bangkok 10400, Thailand; Department of Entomology, Cornell University, Ithaca, NY 14853, United States; Department of Molecular Biology and Genetics, Cornell University, Ithaca, NY 14853, United States; Department of Biological Sciences, University of South Carolina, Columbia, SC 29208, United States; Department of Life Sciences, Imperial College London, Ascot SL5 7PY, United Kingdom

**Keywords:** mosquito, mating behavior, pickpocket, chemosensation, functional genetics

## Abstract

The molecular basis of mating behavior in the important disease vector mosquito, *Aedes aegypti*, remains poorly characterized. We investigated the functional role of a pickpocket gene, *ppk315*, in male mating behavior using both RNAi-mediated knockdown and CRISPR/Cas9 approaches. Behavioral assays revealed that RNAi-treated males (dsPPK315) made fewer mating attempts, were less responsive to female acoustic cues, and were less likely to achieve copulation, though their latency to initiate contact when attempts were made was comparable to controls. Males with a CRISPR/Cas9-induced disruption to *ppk315* exhibited reduced success in inseminating multiple females, consistent with previous reports from RNAi knockdown males, ruling out off-target effects as the source of behavioral changes. In contrast to the results of behavioral assays with RNAi, *ppk315* mutant males (*ppk315*^−/−^) attempted copulation as frequently as wild-type males (*ppk315^+/+^*) but were slower to contact females. Despite these impairments in one-on-one interactions, both dsPPK315 and *ppk315*^−/−^ males displayed normal mating success under competitive swarm-like conditions, potentially due to the socially facilitated activation of mating behavior. Collectively, our findings support a role for *ppk315* in the initiation of mating behaviors via sensory detection, with context-dependent consequences for reproductive success.

## Introduction

The yellow fever mosquito, *Aedes aegypti,* is an important vector of several viruses of public health importance, such as dengue and chikungunya, and is the target of many control campaigns. Over 5 million cases of dengue virus alone were reported in 2023, with that number surpassed in 2024 (Venkatesan 2024). With increasing global temperatures, the transmission of viruses spread by *Ae. aegypti* is predicted to increase in both tropical and temperate regions (Delrieu et al. 2023). In light of widespread *Aedes* resistance to the main classes of chemical insecticides (Schmidt et al. 2024), new control tools are urgently needed.

Many new and developing mosquito control tools involve the mass-release of male mosquitoes, which are either sterile and thus suppress the wild population or are engineered to produce offspring with reduced capacity to transmit disease agents. Both of these approaches require males reared in captivity to successfully mate with wild females upon release. A greater understanding of mating behavior, sexually selected traits, and their genetic determinants will be crucial in developing and increasing the efficacy of these control tools (Cator et al. 2021).

In a recent study, we identified several high-confidence candidate genes with variants that responded to the presence or absence of precopulatory sexual selection, suggesting a role for these genes in sexually selected traits (Wyer et al. 2023). One of these candidate genes was *pickpocket315* (*ppk315*, AAEL000863). *Pickpocket* (*ppk*) genes were first described in the nematode *Caenorhabditis elegans* (Garcia-Añoveros et al. 1995) and belong to a superfamily of Degenerin/ENaC channel genes. Previous research, mostly in the genus *Drosophila,* has implicated *ppk* genes in a range of processes such as the perception of pain (Hwang et al. 2007; Boiko et al. 2012), larval locomotory behavior (Ainsley et al. 2003; Xu et al. 2004), and negative regulation of feeding behavior (Wegman et al. 2010). Furthermore, evidence from multiple studies supports the involvement of *ppk* genes in mating. For instance, *ppk25* is expressed in neurons that detect female-specific pheromones in males, and the absence of *ppk25* in females inhibits their receptivity to males (Vijayan et al. 2014). *ppk23* and *ppk29* are crucial for inhibiting male courtship toward other males and promoting courtship toward females (Thistle et al. 2012). *ppk23* is also involved in the male response to female aphrodisiac pheromone 7,11-heptacosadiene (Toda et al. 2012; Seeholzer et al. 2018).

The role of *ppk* genes is not well understood in *Ae. aegypti*, which is predicted to have 31 *ppk* genes distributed across the genome (Matthews et al. 2018). While bulk and single-cell transcriptomic data have described the tissue-specific expression of *ppk* genes (Matthews et al. 2016, 2018; Goldman et al. 2025), functional characterization has been performed for only a handful of these genes, with a phenotype identified in one: *ppk301*. This gene is found in sensory neurons responding to water and high salt levels, and *ppk301* genetic mutants exhibit changes in egg-laying behaviors (Matthews et al. 2019).

Our previous behavioral screen of *ppk315*-depleted mosquitoes using RNAi identified that depleted males had deficits in their ability to locate and inseminate females in a confined space and in the absence of competition (Wyer et al. 2023). The behavioral mechanisms that underpin the reduced insemination success phenotype of *ppk315*-depleted males observed in our previous study are currently unknown and the possibility of additional effects on male mating behavior was not explored. In addition, while the RNAi-mediated gene silencing approach has been used to great effect to identify genes involved in mosquito physiology and behavior (Blandin et al. 2002; Attardo et al. 2006; Xi et al. 2008; Thailayil et al. 2011; Erdelyan et al. 2012), the occurrence of off-target effects in RNAi methods, such as silencing of nontarget mRNAs (Carthew and Sontheimer 2009), introduces a potential lack of specificity that may generate false positives in a screen.

Here, we expand our functional characterization of *ppk315* to determine its impacts on free-flight mating interactions, phonotactic responses, and competitive mating interactions. In addition to RNAi silencing, we used CRISPR/Cas9 genome editing to generate a genetic *ppk315* mutant. Male mosquitoes with *ppk315* disrupted by CRISPR/Cas9 show reduced insemination success relative to control males, confirming that previously observed insemination success deficits of *ppk315*-depleted males are not a product of off-target RNAi effects. In one-on-one interactions, both *ppk315-*depleted and mutant males exhibited differences in mating interactions, with depleted males being less likely to attempt to mate with females and mutant males taking longer to initiate mating attempts compared to controls. Intriguingly, differences are not observed when *ppk315-*deficient males directly competed with control males for females. In scenarios in which they were held with control males, depleted and mutant males appear to behave similarly. These additional data further support a role for *ppk315* in mating behavior and suggest that, as in other Dipterans, *ppk315* could be involved in male perception of cues which aid in the initiation of mating responses.

## Methods

### Mosquito rearing

RNAi experiments were all performed using a recently established (F8-10) strain of *Ae. aegypti* originating from Kamphaeng Phet Province (KPP), Thailand, hereafter referred to as the “Thai” strain (Qureshi et al. 2019). Eggs were hatched under a vacuum, provided with 0.1 mg of ground fish food and held overnight at 27 °C, 75% RH. First instar larvae were sorted into trays of 500 larvae/1 L water and provided fish food pellets *ad libitum.* Pupae were sorted by sex and allowed to emerge in separate cages. All mosquitoes were maintained on 10% w/v sucrose. Experimental blocks were derived from independently-hatched cohorts.

### dsRNA synthesis and injection

dsRNA targeting *ppk315* was synthesized as described previously (Wyer et al. 2023). Male Thai mosquitoes 0–2 d posteclosion were anesthetized on ice and injected intrathoracically with dsRNA (500 ng dsRNA in a 69 nL volume per mosquito) using the Nanoject II microinjector (Drummond Scientific) and borosilicate glass capillary needles. Mosquitoes were allowed two days to recover postinjection and provided with 10% sucrose *ad libitum*. The extent of gene expression knockdown was assessed by qPCR (Supplementary Fig. 1).

### Ppk315 mutant generation via CRISPR

We generated a mosquito line with a disruptive (361 bp) insertion in the coding sequence of *ppk315* (AAEL000863) by CRISPR/Cas9-based gene editing according to the methods outlined in Kistler et al. (2015) (Supplementary Fig. 3). Sites were identified for cleavage using the program CHOPCHOP (Labun et al. 2016). Three guide RNAs (gRNAs) were synthesized *in vitro* using the MEGAScript kit (Thermo Fisher Scientific) from DNA generated from a template-free PCR consisting of a primer targeting the Cas9 cut site with a T7 promoter and a universal reverse primer (sequences listed in Supplementary Table 1) and cleaned with MEGAClear (Thermo Fisher Scientific) as per the manufacturer's instructions. gRNAs were further purified through a precipitation and ethanol wash (Kistler et al. 2015) and then combined with commercially acquired Cas9 protein (PNA Bio) before injection into Thai strain embryos by the University of Maryland Insect Transformation Facility (https://www.ibbr.umd.edu/facilities/itf).

G0 females were backcrossed to Thai wild-type males, blood fed, and allowed to oviposit. Genomic DNA (gDNA) from G0 females was extracted using Puregene reagents (Qiagen), PCR and Sanger sequencing were used to detect insertions in the *ppk315* gene that would result in a premature stop codon (Supplementary Table 1; Supplementary Fig. 3). Eggs from insertion-positive females were hatched and back-crossed for 5 generations. Heterozygous males and females were crossed, and progeny screened for genotype using genomic DNA extracted from a single leg (Smith et al. 2018). Males (*n* = 8) and females (*n* = 6) homozygous for the *ppk315* insertion were crossed together to generate a stable mutant line (*ppk315*^−/−^). A wild-type control line (*ppk315*^+/+^) derived from a backcross of heterozygous individuals was also maintained.

### Insemination success assay with ppk315 mutants

As in the previous study (Wyer et al. 2023), male insemination success was assessed by aspirating a single male mosquito of either the *ppk315*^−/−^ or *ppk315*^+/+^ genetic background into 455 mL cups with 6 virgin Thai females. Mosquitoes were provided 10% sucrose solution *ad libitum* and allowed to mate for 24 h, after which all females were collected, anesthetized on ice, and their spermathecae dissected in 1×phosphate-buffered saline (PBS) and examined under a brightfield microscope for the presence or absence of sperm to determine insemination status. The insemination success of 20 males of each genotype was measured over 2 independent experimental blocks (*n* = 40 males/treatment).

### Single male–female mating interactions in depleted and mutant ppk315 males

To capture any change in the ability of males to locate and orient to females, we observed one-on-one interactions between single males and females. To investigate the impact of depletion, we released a single virgin male 2 dpi dsGFP or dsPPK315 into a 19 cm^3^ Perspex mating cage provided with a 10% sucrose solution the night before the trials to allow males to habituate to mating cages. Trials began the following day when a single 3–5 d old WT Thai female was added to each cage using a mouth aspirator and the number of mating attempts, defined as physical contact between the male and female, were recorded. The time from female release to the first mating attempt was recorded as the contact latency, and whether the pair formed a mating copula (defined by genital contact between the male and female) was recorded (Y/*N*). For those pairs that formed a mating copula, the length of time for which there was genital contact between the male and female was defined as copula duration. The trial was halted after 4 min, or after a mating copula was formed, whichever occurred first. For those trials where the male and female formed a mating copula, the female was removed, anesthetized on ice, and the spermathecae dissected in 1×PBS and examined under a brightfield microscope to confirm that insemination had occurred. All data were recorded by the observer (who was blind to the treatment condition) at the time of the assay and all experiments were performed between 0900 and 1200 h. The experiment was run 3 times, with 20 males for each treatment in the first experimental block, 30 males for each treatment in the second experimental block, and 29 dsGFP males and 30 dsPPK315 males in the third experimental block (159 trials total; 79 dsGFP and 80 dsPPK315).

We also compared the performance of virgin 2-d-old *ppk315*^−/−^ or *ppk315*^+/+^ using the same assay. This experiment was run 3 times, with 20 males for each treatment per experimental block (120 trials total; 60 *ppk315*^−/−^ and 60 *ppk315*^+/+^).

### Phonotactic response of ppk315-depleted males

DsPPK315 or dsGFP male mosquitoes 2-d postinjection were held in 455 mL cups with 10% sucrose solution provided *ad libitum.* Sucrose was removed from the cups at 0800 on the day of the phonotactic assay. Three males from each treatment were aspirated into a 19 cm^3^ plexiglass cage containing a single earphone speaker. Males were allowed 1 min to acclimatize to the cage before a 480 Hz tone (as in Cator and Zanti (2016)) was played through the speaker at maximum volume (∼100 dB) in bouts of 10 s, with 5 s intervals between each bout, for a total of 2 min. Due to the difficulty in observing multiple males in this assay, all data were recorded by watching video playback of each 2-min trial. The video was recorded using a GoPro HERO4 camera mounted on a clamp. Videos were used to record the timing of each contact any one of the males made with the speaker during the trial. A contact was defined by male legs touching the speaker. Whether or not any one male physically contacted the speaker was recorded (Y/N), and the time between the start of the trial and when physical contact was made with the speaker was recorded as the contact latency. The proportion of the 2min trial for which any one male was in flight was determined by watching the video recordings and manually calculating activity time. All experiments were performed between 0900 and 1200.

### Competitive mating assay with ppk315 CRISPR/Cas9 mutants

For easy and immediate identification of individual male genotype following competition experiments, male mosquitoes were marked with different colored fluorescent powders using the “paint method” (eg Lang et al. (2018)). Briefly, virgin 1-d-old males from both the *ppk315*^−/−^ and *ppk315*^+/+^ genetic backgrounds were anesthetized on wet ice for around 2 min. Males were then moved to petri dishes and a 2 cm water color brush doused in 1 of 2 dust colors (yellow or pink: Swanda Inc., Stalybridge, United Kingdom) was used to sprinkle dust onto males. Two males from each genotype were then aspirated into 19 cm^3^ transparent plexiglass cages for the competitive mating experiment and provided with cotton wool soaked in a 10% sucrose solution. The following day at 0900, a single Thai female was aspirated into the cage. When copula formation was observed, the pair was aspirated out of the cage and the male and female were transferred to separate labeled cups. A copula was counted when the male was observed to clasp its genitalia to the female genitalia. The trial was halted after the observed copula, or after 4 min, whichever occurred first. The time of the copula and its duration were recorded. The mated females were later anesthetized on ice and their spermathecae dissected in 1×PBS and examined under a brightfield microscope for the presence or absence of sperm to confirm insemination status. Males were examined under a dissecting microscope to view the fluorescent dust, thereby identifying the genotype. The experiment was repeated 3 times with 20, 27, and 26 trials per experimental block, respectively (total trials = 73).

### Statistical analysis

All statistical analyses were performed using R version 4.3.3. Data were checked for normality using the Shapiro–Wilk test and analyzed accordingly.

Experiments examining the effect of *ppk315* transcript knockdown or genetic disruption on single male–female mating interactions were assessed with linear mixed models (LMM) and the R packages “lme4” and “afex” with copulation duration, time to first attempt, or number of mating attempts as the response variables, treatment (either dsPPK315 or dsGFP for RNAi treatments, or *ppk315*^−/−^ or *ppk315*^+/+^ for CRISPR/Cas9 treatments) as a fixed factor and experimental block as a random factor. Contact latency was assessed using a Cox proportional hazards regression model using the “survival” and “survminer” packages. The predictor variable was the RNAi treatment (dsPPK315 or dsGFP), the response variable was latency to first attempt, with individuals that did not attempt within the observation period treated as right-censored, and the experimental block was a random factor.

The effect of *ppk315* depletion on the phonotactic response of males was analyzed using linear mixed models with contact latency or activity time as the response variables, treatment (dsPPK315 or dsGFP) as a fixed factor and experimental block as a random factor. To determine if *ppk315* expression affected the likelihood of a male making contact with the speaker, we used a generalized linear mixed model (GLMM) with a binomial distribution in which the RNAi treatment was the predictor variable and whether or not the male contacted the speaker (recorded as Y/N) as the response variable, with experimental block fit as a random effect.

To determine whether the proportion of matings achieved by *ppk315*^−/−^ or *ppk315*^+/+^ differed from the null expectation of 50% in the competitive mating assay, we used a GLMM with a binomial distribution in which the genotype of the male mating in each trial was the response variable with experimental block fit as a random effect. In this model, a significant intercept indicates a difference between genotypes in the proportion of matings achieved. We square root-transformed the time to copula (s) and tested for an effect of male genotype using an LMM with the genotype of the successful male as a fixed factor and experimental block as a random factor.

The effect of genotype (*ppk315*^−/−^ or *ppk315*^+/+^) on a male's ability to mate up to 6 virgin females over a 24-h period during the insemination success assay was assessed using an LMM. The model included the number of females inseminated as the response variable and genotype as the predictor variable, with a random effect of experimental block included.

## Results

### Male behavior in single male–female interactions in *ppk315-*depleted males

**Fig. 1.**
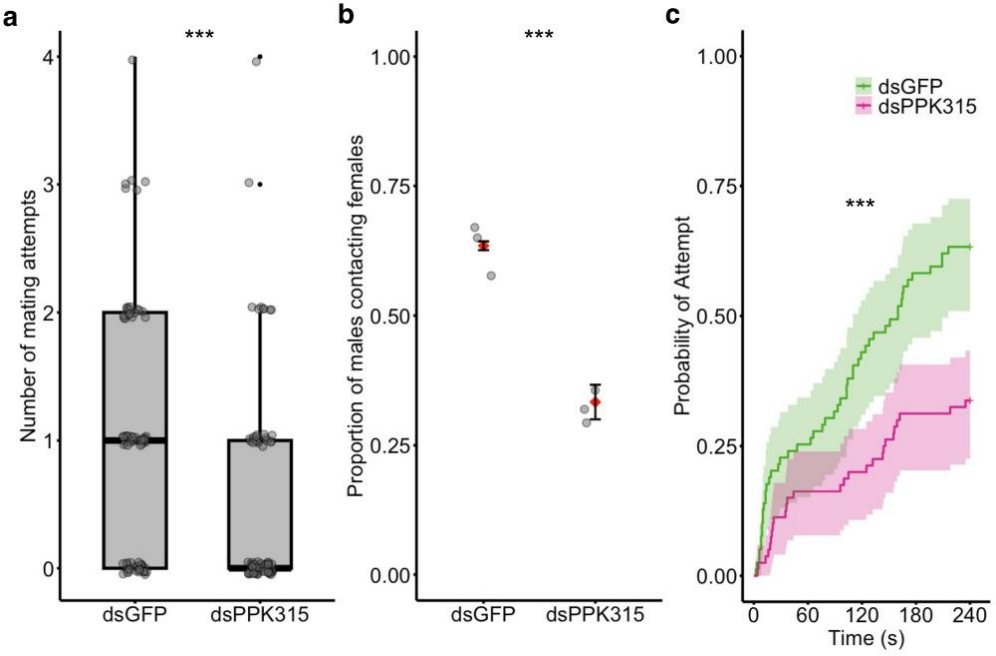
Single male–female mating interactions following RNAi targeting *pickpocket 315* (dsPPK315) and control gene GFP (dsGFP). a) Number of mating attempts made in the 4-min trial. Data from individual males are shown as black circles and bold black lines indicate the median. b) Proportion of males that contacted females for each individual experimental block shown in black circles, overall mean from 3 experimental blocks denoted by red triangles. Error bars represent ±1 SE. c) Cumulative probability of males making contact with females over time. Inverted Kaplan–Meier survival curves represent the probability of a male mating attempt over the course of the trial for the 2 treatments: dsGFP in green and dsPPK315 in pink. Shaded areas represent 95% C.I. Data are right-censored for males who did not attempt contact during the observation period. A total of *n* = 70 dsGFP males and *n* = 80 dsPPK315 males were assayed across 3 experimental blocks. Three stars (***) indicates a significant difference at *P* < 0.001 between the dsPPK315 test treatment and GFP control treatment.

In observations of one-on-one interactions, dsPPK315 males made fewer attempts to mate with females than dsGFP controls (Fig. 1a; dsGFP mean no. of attempts = 1.00 ± 0.110 SE, dsPPK315 mean no. of attempts = 0.488 ± 0.091 SE). This resulted in depleted males being less likely than control males to contact a female during the trial (Fig. 1b; binomial GLM: χ²(1) = 14.10, *P* < 0.001), with the male treatment having a significant effect on the latency to attempt mating (Fig. 1c; *P* < 0.001). However, if dsPPK315 males did contact a female, they exhibited a similar latency between the start of the trial and the time of first contact (dsGFP mean contact latency = 86.5 s ± 12.4 SE, dsPPK315 mean contact latency = 89.4 s ± 15.4 SE) and were as likely to form a copula (binomial GLM; χ²(1) = 2.19, *P* = 0.139). In interactions that culminated in copula formation, females were as likely to have been successfully inseminated if they had formed a copula with either dsPPK315 males or dsGFP males (binomial GLM; χ²(1) = 2.19, *P* = 0.139).

### Phonotactic response in *ppk315-*depleted males

To parse whether the difference in male response to females was due to the activity of those females, we used a phonotactic assay in which we could standardize the signals males received and compare their response. When we measured how these males responded to controlled stimuli in a phonotactic assay, we found that while males with depleted expression of *ppk315* spent a similar amount of time in flight during the trial (c. S2C; LMM: *F*_1, 56.20_ = 0.648, *P* = 0.424), they were less likely to make contact with a speaker producing frequencies similar to that of females than control males (Supplementary Fig. 2a; Binomial GLM: χ²(1) = 4.40, *P* = 0.0359). If dsPPK315 males did respond to stimuli, they did so with a similar latency to dsGFP males (Supplementary Fig. 2b; LMM: *F*_1, 24_ = 0.064, *P* = 0.803).

### Male insemination success and courtship in *ppk315* genetic mutants

**Fig. 2.**
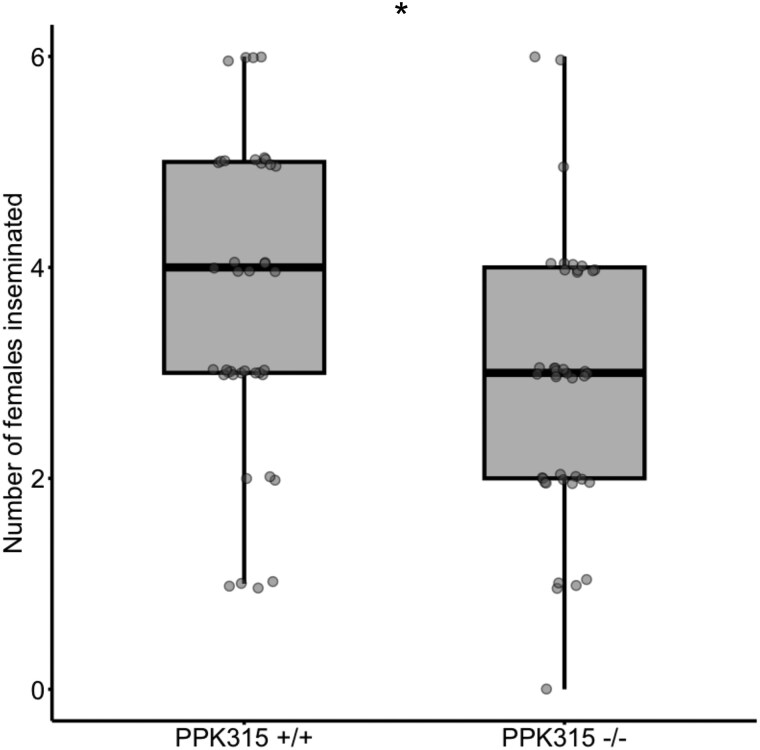
The effect of CRISPR/Cas9-induced mutation of *ppk315* on male insemination success. Number of virgin females inseminated by single males in 24 h in 455 mL. cups. Bold black lines indicate the median. A total of *n* = 40 *ppk315*^−/−^ males and *n* = 39 *ppk315*^+/+^ males assayed across 2 experimental blocks. One star (*) indicates a significant difference at *P* < 0.05 between the *ppk315*^−/−^ test treatment and *ppk315*^+/+^ control treatment.

We previously reported a reduced insemination success in *ppk315*-depleted males in a 24 h period in the absence of competitors (Wyer et al. 2023). Here, we observed a similar outcome for mutant males with the coding sequence of *ppk315* disrupted using CRISPR/Cas9 (Supplementary Fig. 3); *ppk315*^−/−^ males inseminated a mean of 2.90 (±0.205 SE) females in 24 h, significantly fewer than the 3.67 (±0.233 SE) females inseminated by *ppk315*^+/+^ males (Fig. 2; LMM: *F*_1, 76.01_ = 6.22, *P* = 0.0148).

**Fig. 3.**
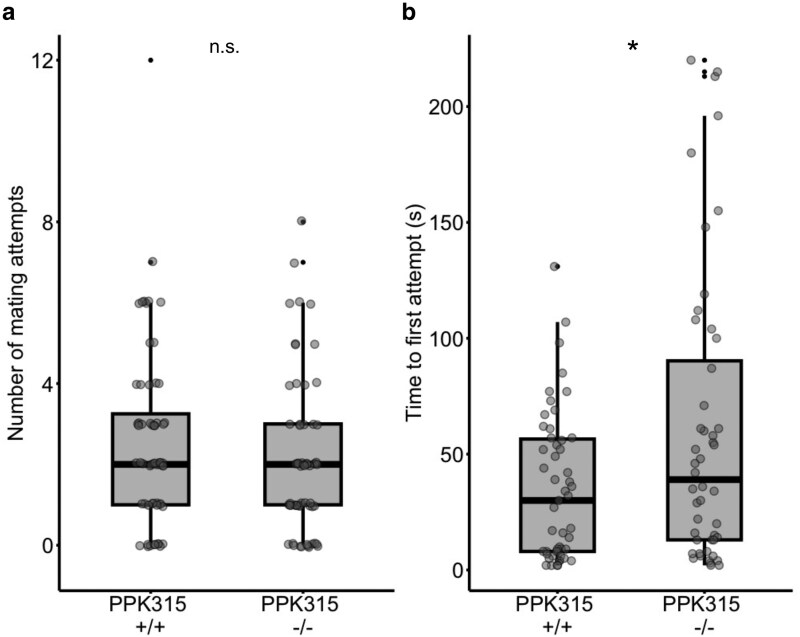
One-on-one mating interactions between *ppk315* CRISPR/Cas9 males and wild-type females. a) Number of mating attempts recorded during interactions between *ppk315*^−/−^ and *ppk315*^+/+^ males presented with a wild-type female. Data from individual males in black dots (*ppk315*^−/−^  *n* = 60, ppk*315*^+/+^  *n* = 60) and bold black lines indicate the median. b) Time between the start of the trial and the first observed mating attempts. Data from individual males in black dots (*ppk315*^−/−^  *n* = 60, *ppk315*^+/+^  *n* = 60), bold black lines indicate the median. One star (*) indicates a significant difference at *P* < 0.05 between treatments using a linear model.

To confirm that the effects of RNAi-mediated *ppk315* expression reduction on single male–female interactions were not merely due to off-target effects, we repeated this experiment using *ppk315* genetic mutants generated using CRISPR/Cas9. As in the RNAi experiments, we observed differences in one-on-one mating interactions between *ppk315* mutants with virgin wild-type females compared to controls (Fig. 3). However, unlike the dsPPK315 males, *ppk315*^−/−^ males attempted to mate with these females a similar number of times (Fig. 3a; LMM: *F*_1, 116_ = 1.726, *P* = 0.192) and were as likely to inseminate females as the *ppk315*^+/+^ control males (binomial GLM: χ²(1) = 0.32, *P* = 0.572). However, they also exhibited a greater latency between the start of the mating trial and when they made their first attempt (Fig. 3b; LM: *F*_1, 97_ = 6.512, *P* = 0.0123).

**Fig. 4.**
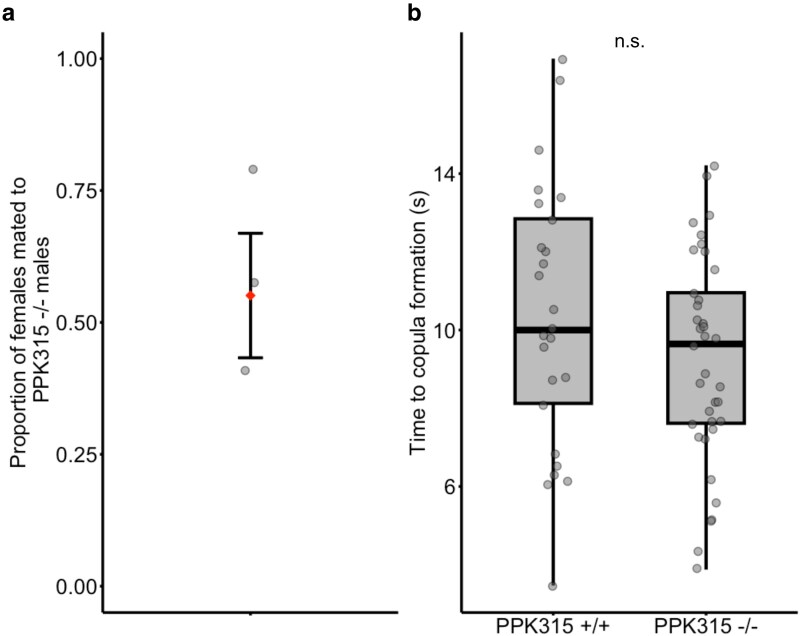
Outcome of mating competition between *ppk315*^−/−^ and *ppk315*^+/+^ males presented with a wildtype female. a) Proportion of matings in which females mated with *ppk315*^−/^males (*n* = 62 competition trials in total across 3 experimental blocks). Error bars represent ±1 SE. b) Square root-transformed time (seconds) between the start of the trial and the time the copula started. Data from individual winning males are shown by black dots (*ppk315*^+/+^  *n* = 25, *ppk315*^−/−^  *n* = 37), bold black lines indicate the median.

Interestingly, when *ppk315^−/−^* males competed directly against *ppk315^+/+^* males they were as likely to mate with wild type females (Fig. 4a; EMM = 0.581, SE = 0.0982) and the previously reported differences in attempt number and latency observed in one-on-one interaction in males with interrupted *ppk315* expression (Figs. 1 and 3) were not observed (Fig. 4b; *ppk315^+/+^* EMM = 10.56, SE = 1.05; *ppk315^−/−^* EMM = 9.68, SE = 1.03).

## Discussion

Despite the public health impact of *Ae. aegypti* and the role of male mating success in control strategies, the molecular underpinnings of the complex precopulatory interactions between male and female mosquitoes remain largely uncharacterized (Su et al. 2020; Wang et al. 2024). In this study, we build upon previous work demonstrating that *ppk315*-depleted males have reduced insemination success. Using 2 different methods to disrupt *ppk315*, our results further support a role for this gene in male mating behavior.

While Wyer et al. (2023) measured a male's ability to mate in the absence of competitors and with a surplus of females, the assays deployed here allowed us to detect more nuanced differences in phenotype. We found that, when alone with a female, dsPPK315 males made fewer attempts to mate and were less likely overall to form a successful copula (Fig. 1). However, those males that did make an attempt to mate with a female, did so just as quickly as control dsGFP males. This result suggests that males with reduced expression of *ppk315* may be less adept at perceiving female cues that would initiate a mating attempt. Indeed, a recent single-nucleus transcriptomic atlas of *Ae. aegypti* tissues (Goldman et al. 2025) revealed that *ppk315* is expressed in a distinct subpopulation of cells in the maxillary palps, an important site of sensory perception (Bohbot et al. 2014). However, once the female is detected, their ability to locate and contact a female was not impaired. The variation in phenotype observed between individuals may reflect the incomplete silencing of *ppk315* mRNA induced by RNAi. Some injected males may still express sufficient levels of the gene to exhibit a comparable phenotype to the controls.

While RNAi-mediated gene silencing has proven a valuable tool in the reverse genetics studies of mosquito phenotypes (Rono et al. 2010; Olmo et al. 2018), some studies have identified inconsistencies with gene knockdown efficiency (Figueiredo Prates et al. 2024) and potential off-target effects (Chen et al. 2021), which can generate false-positives in RNAi screens (Carthew and Sontheimer 2009). In this study, we confirmed that the reduced insemination success phenotype observed in males with reduced expression of *ppk315* via RNAi-mediated gene silencing is also evident in males with a CRISPR/Cas9-mediated mutation to the gene resulting in a premature stop codon that is expected to lead to loss of function. This suggests that the results observed in dsPPK315 males were unlikely to be due to off-target effects (though whole-transcriptome analysis would be required to eliminate this possibility entirely) and further implicates *ppk315* as important for male mating success.

In one-on-one interactions with females, *ppk315^−/−^* males make a comparable number of mating attempts as *ppk315^+/+^* males. However, *ppk315^−/−^* males are slower to make initial contact with females than *ppk315^+/+^* males. This discrepancy may reflect important differences between the two gene silencing methods; by silencing the gene at the DNA level, CRISPR-generated mutants do not express *ppk315* at any life stage, whereas the RNAi method is performed on adult mosquitoes and transiently (and incompletely) reduces gene expression at the mRNA level. It is plausible that these 2 approaches may generate different results in males, eg, the CRISPR mutants might have alterations in their nervous system due to the loss of *ppk315* throughout development, or there could be upregulation of compensatory genes during development that mask the phenotypic effects of CRISPR-generated mutations (Salanga and Salanga 2021). Future work could measure expression levels of other *ppk* genes with similar functional domains in mutant lines to explore this possibility. Perhaps the total lack of gene expression in *ppk315^−/−^* males is responsible for the relatively high contact latency, whereas the residual mRNA expression in dsPPK315 males is sufficient to maintain a comparable contact latency to control males. Performing tissue- and stage-specific transgenic manipulation of this gene would clarify its role in mating behavior (Riabinina et al. 2015).

Finally, we observed that when in competition with other males, there was no effect of *ppk315* mutation on the likelihood of mating success. One possible explanation is that deficits that we observed in the response time to females in *ppk315^−/−^* males may not be sufficient to disadvantage them in competitive interactions. This, however, seems unlikely, as the mean response time of *ppk315^−/−^* (60.6 ± 7.18 SE) was almost double that of the *ppk315^+/+^* males (35.1 ± 6.96 SE) and mating swarms are intense arenas of scramble competition (Cator et al. 2021), where precedence in recognition of females is likely critical. It could also be that the slower response time observed in these mutants is compensated for by a different response, such as accuracy, which we did not measure in our assays. For example, mutant males might be slower to respond but better at intercepting females or better at delivering courtship cues when they do.

An alternative explanation for the lack of an effect of *ppk315* knockout on male mating success in competition, as opposed to the significant effects observed in one-on-one interactions with females, is that the *ppk315* phenotype is context-dependent. In their natural context, these insects mate in the presence of multiple males. Though it is known that small groups of males aggregate in the vicinity of a vertebrate host awaiting mating opportunities (Cator et al. 2011; Oliva et al. 2014), the specific cues that stimulate male mating responses within swarms remain unknown. Male insects of several species have been shown to modulate mating-associated behaviors with such factors as circadian timing (Wang et al. 2021) or social context, often intensifying courtship displays in the presence of conspecific males (Bretman et al. 2009, 2011, 2012; Callander et al. 2013; Setoguchi et al. 2015). The ability of *ppk315^−/−^* males to perform comparably to wild type males when multiple rivals are present suggests that males may be sufficient to stimulate each other to respond to females even when *ppk315* is not expressed. This is supported by our results from the phonotactic assay showing that dsPPK315 males were no less active than dsGFP males, but less likely to respond to female acoustic cues (Supplementary Fig. 2). However, it should be noted that the assay cannot differentiate between males that specifically fail to respond to female flight tones and those that do not respond to acoustic stimuli at all, as responses to unrelated frequencies were not tested. Future work could include specific probing of the acoustic sensitivity of dsPPK315 and mutant males.

Both knockout and knockdown genetic manipulations are powerful tools, but, at least in this context, not entirely sufficient to dissect gene function. The consistent thread across this series of experiments is that *ppk315* impacts the ability of single males to successfully mate. Although *ppk315* expression has been detected in both the testes and the maxillary palps of male *Ae. aegypti* (Matthews et al. 2016, 2018; Goldman et al. 2025), its functional role remains unclear. Expression in the palps is likely most relevant to the behavioral phenotypes observed here, as these appendages are well-established sensory organs in mosquitoes, responsible for detecting CO₂ and components of human odor (Athrey et al. 2021; Riabinina et al. 2015; Syed & Leal 2007). Recent snRNA-seq data show that *ppk315* is enriched in a distinct subpopulation of palp cells marked by the transcription factor Sox100B, where it is co-expressed with other *ppk* genes such as *ppk313* and *ppk314* (Goldman et al. 2025). The specific contribution of *ppk315* to these cells is unknown, and our ability to speculate is limited by the absence of clear transcriptional orthologs outside of the genus. Nonetheless, studies in *Drosophila* suggest that *ppk* subunits can act as amplifiers of sensory signals downstream of olfactory receptors (Ng et al. 2019). Functional studies examining the activity of these cell types, as well as the potential redundancy or cooperative roles of co-expressed *ppk* genes, will be essential to clarify the contribution of *ppk315* to male mating behavior.

In conclusion, our study provides strong evidence that *ppk315* influences male *Ae. aegypti* mating behavior, particularly in scenarios where perception of female cues is crucial. Using both RNAi knockdown and CRISPR/Cas9 knockout approaches, we show that disrupting *ppk315* impairs mating success, likely through context-dependent effects on sensory perception. This work advances our understanding of mosquito mating behavior and highlights *ppk315*, and potentially other *ppk* family genes, as promising targets for further investigation and manipulation. These genes may play a role in the development of reproductive control strategies—either by serving as targets for genetic manipulation in released populations or as molecular markers for monitoring mating competence in captive mosquito lines.

## Supplementary Material

jkaf297_Supplementary_Data

## Data Availability

All data and strains presented in this study are available upon request from the corresponding author. Raw data is available in Supplementary Data S1. Supplemental material available at G3 online.
