# Supplementary material for: Pickpocket315 affects male mating behavior in the yellow fever mosquito Aedes aegypti
Source: G3 (Bethesda). 2025 Dec 10;16(2):jkaf297. doi: 10.1093/g3journal/jkaf297 (PMC12869071; doi:10.1093/g3journal/jkaf297)
Supplement: jkaf297_Supplementary_Data [file jkaf297_supplementary_data.zip › Figure_S1_G3-2025-406212.docx]

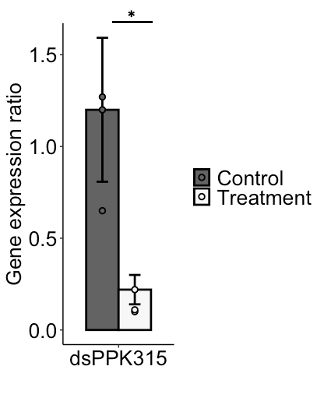
***Figure S1.* Efficacy of *ppk315* gene silencing using RNAi in *Ae. aegypti* males.** Gene expression ratio of dsPPK315 with respect to a dsGFP control. Bars show mean and two standard errors of gene expression ratio of gene of interest, *pickpocket 315* (AAEL000863) relative to ribosomal S7 housekeeping gene from three experimental blocks (individual blocks plotted as dots) of 20 whole male mosquitoes. P-values less than 0.05 are marked with one star (*).
