# Supplementary material for: Pickpocket315 affects male mating behavior in the yellow fever mosquito Aedes aegypti
Source: G3 (Bethesda). 2025 Dec 10;16(2):jkaf297. doi: 10.1093/g3journal/jkaf297 (PMC12869071; doi:10.1093/g3journal/jkaf297)
Supplement: jkaf297_Supplementary_Data [file jkaf297_supplementary_data.zip › Figure_S2_G3-2025-406212.docx]

***Figure S2*. Male phonotactic response to artificial female flight tones following RNAi**. **a)** Proportion of males contacting speaker playing a female flight tone during trial. Circles represent the proportion of males contacting speaker from an individual experimental block. The triangles depict the mean of the three experimental blocks, bars are standard error. **b)** Boxplot showing the latency to speaker contact in seconds, with only males that contacted the speaker included. For the dsGFP treatment the sample sizes were n = 6 and n = 11 for experimental blocks 1, 2, respectively, whilst no observations were recorded for block 3. For the dsPPK315 treatment the sample sizes were n = 2 and n = 7 for experimental blocks 1 and 2, respectively, whilst no observations were recorded for block 3. Circles represent the contact latency times for individual males. **c)** Boxplot showing the number of seconds that individual males were in flight during the assay. Dots are the amount of time (in seconds) spent in flight for individual cages. One star (*) indicates a significant difference between the *pickpocket315* test treatment and GFP control treatment at a p-value <0.05. Three experimental blocks were performed in total. For both the dsGFP and the dsPPK315 treatments there were n = 10, n = 15, and n = 5 males assayed for blocks 1, 2 and 3, respectively.
