# Supplementary material for: Pickpocket315 affects male mating behavior in the yellow fever mosquito Aedes aegypti
Source: G3 (Bethesda). 2025 Dec 10;16(2):jkaf297. doi: 10.1093/g3journal/jkaf297 (PMC12869071; doi:10.1093/g3journal/jkaf297)
Supplement: jkaf297_Supplementary_Data [file jkaf297_supplementary_data.zip › Figure_S3_G3-2025-406212.docx]

***Figure S3.* Generation and confirmation of *Ae. aegypti ppk315* CRISPR mutant lines. a)** Diagram of the wild type *Ae. aegypti* *ppk315* genomic locus. Guide RNA sites used for generating mutants are indicated with vertical red lines and location of genotyping primers indicated with blue arrows. **b)** Diagram of the *ppk315* mutant allele generated by insertion of 361 bp using gRNAs (sequences in Table S1). **c)** Sequence analysis using gel electrophoresis. Gel shows PCR amplicons for the wild type (+/+) and mutant (-/-) locus **d)** Amino acid alignments of wildtype (+/+) and mutant (-/-) alleles. Premature stop codons are indicated with an asterisk.
