# Supplementary material for: Pickpocket315 affects male mating behavior in the yellow fever mosquito Aedes aegypti
Source: G3 (Bethesda). 2025 Dec 10;16(2):jkaf297. doi: 10.1093/g3journal/jkaf297 (PMC12869071; doi:10.1093/g3journal/jkaf297)
Supplement: jkaf297_Supplementary_Data [file jkaf297_supplementary_data.zip › Table_S1_G3-2025-406212.docx]

***Table S1.*** Primer sequences for generation of CRISPR/Cas9 *ppk315* lines

| **Description** | **Sequence** |
| --- | --- |
| Universal primer | 5’ AAA AGC ACC GAC TCG GTG CCA CTT TTT CAA GTT GAT AAC GGA CTA GCC TTA TTT TAA CTT gct att tct agc tct aaa ac 3’ |
| Guide 1 primer | 5’ GAA ATT AAT ACG ACT CAC TAT A **GGA CTT CTT CTC ACA TAC GT** gtt tta gag cta gaa ata gc 3’ |
| Guide 2 primer | 5’ GAA ATT AAT ACG ACT CAC TAT A **GGG CAC GAG AAA TCT ACC** **GA** gtt tta gag cta gaa ata gc 3’ |
| Guide 3 primer | 5’ GAA ATT AAT ACG ACT CAC TAT A **GGG TTT CAC ACG GTA CCA GC** gtt tta gag cta gaa ata gc 3’ |
| Genotyping primer (forward) | 5’ GCG ATT TCA TCT CGC TAC G 3’ |
| Genotyping primer (reverse) | 5’ TTT CGG TTC TTC TGA TTC GG 3’ |
